# Development of a Machine Learning Model Integrating Pathomics and Clinical Data to Predict Axillary Lymph Node Metastasis in Breast Cancer: A Two‐Center Study

**DOI:** 10.1002/cnr2.70302

**Published:** 2025-08-31

**Authors:** Long Wang, Fanli Qu, Ping Wen, Yu Luo, Huan Zhang, Shanqi Li, Xuedong Yin, Yulan Zhao, Xiaohua Zeng

**Affiliations:** ^1^ Department of Breast Cancer Center Chongqing University Cancer Hospital Chongqing China; ^2^ Department of Breast Cancer Center, Chongqing University Cancer Hospital, School of Medicine Chongqing University Chongqing China; ^3^ Department of Breast and Thyroid Surgery The First Affiliated Hospital of Chongqing Medical University Chongqing China; ^4^ Department of Medical Insurance Chongqing University Cancer Hospital Chongqing China

**Keywords:** axillary lymph node metastasis, breast cancer, machine learning, nomogram model, pathomics

## Abstract

**Background:**

Accurately assessing the status of axillary lymph nodes (ALNs) is essential for devising optimal surgical plans and making informed treatment decisions in breast cancer (BC) patients.

**Aims:**

This study aims to develop an innovative nomogram based on pathomics to preoperatively predict ALN metastasis (ALNM) in BC.

**Methods and Results:**

Our study performed a retrospective analysis on digital hematoxylin and eosin (H&E)‐stained images obtained from 407 patients across two institutions who were allocated into a training cohort (TC; *n* = 203), an internal validation cohort (IVC; *n* = 136), and an external validation cohort (EVC; *n* = 68). Initially, the Mann–Whitney *U*‐test and Spearman's rank correlation coefficient were utilized for feature selection, employing the least absolute shrinkage and selection operator (LASSO) regression for further refinement. For the evaluation of the predictive value of ALNM and other clinicopathological factors, we deployed both univariate (ULR) and multivariate (MLR) logistic regression analyses. Among the six machine learning (ML) algorithms, logistic regression, which demonstrated the highest area under the curve (AUC) value, was employed to establish the final nomogram model. The nomogram reliability and stability were assessed by analyzing the AUC of the receiver operating characteristic (ROC) curve, decision curve analysis (DCA), and calibration plots. MLR analysis demonstrated estrogen receptor (ER), human epidermal growth factor receptor 2 (HER2), tumor size, and pathomics features as independent ALNM predictors. The nomogram demonstrated that the AUC in the IVC (0.783) surpassed that of the Path‐score model (0.698) (DeLong test, *p* = 0.008558). Similarly, in the EVC, the nomogram surpassed the clinical model regarding AUC (0.738 vs. 0.574; DeLong test, *p* = 0.00494). Additionally, DCA analysis indicated a net clinical benefit associated with the nomogram.

**Conclusion:**

Our study demonstrates the effectiveness of pathomics features in predicting ALNM in BC patients. Furthermore, the pathomics‐based nomogram offers a valuable tool for personalized treatment planning in this patient population.

## 1 Introduction

Breast cancer (BC) is one of the most prevalent malignancies and a leading cause of cancer‐related deaths among women worldwide [1]. Patients with positive axillary lymph node (ALN) status are typically classified as high‐risk and often require neoadjuvant chemotherapy (NAC). The Z0011 trial reported that for patients with clinical T1/T2 BC and one or two metastatic sentinel lymph nodes (SLNs), indicating a low metastatic burden, omitting ALN dissection (ALND) in favor of breast‐conserving surgery and systemic therapy yielded comparable survival rates to those undergoing ALND [2, 3]. The SLN biopsy (SLNB) has become the standard due to its reduced risk of severe complications compared to ALND. However, limitations of SLNB include extended anesthesia times due to waiting for intraoperative pathology results and potential upper limb complications like numbness, paresthesia, and lymphedema [4, 5, 6]. Therefore, accurate assessment of ALN status and the extent of metastatic spread remain critical for determining prognosis and guiding treatment decisions in BC management.

In clinical practice, there is a growing emphasis on developing noninvasive methods to assess ALN status in BC patients. Previous studies using mammography, ultrasound, and MRI have explored radiomics analysis of primary malignancies to predict ALN status and metastatic burden. These studies have shown promising results, with AUC scores ranging from 0.64 to 0.89 for ALN status and from 0.74 to 0.79 for metastatic burden [7, 8, 9]. To improve predictive power beyond radiomics alone, some studies have incorporated ultrasound or MRI findings of ALNs or clinicopathological features into nomograms [10]. However, radiomics provides only a macroscopic, in vitro view of the tumor, which does not fully capture the specific characteristics of ALNs. Histopathology images analyzed with machine learning (ML) offer another crucial source of medical information, aiding in risk stratification, prognosis, and predicting the effectiveness of adjuvant chemotherapy. Pathomics, distinct from radiomics, offers microscopic insights into the tumor microenvironment (TME), deepening our understanding of tumor heterogeneity and potentially improving the predictive accuracy of current models [11, 12, 13].

Hematoxylin and eosin (H&E)‐stained slide analysis remains a cornerstone of BC diagnosis. Recently, the concept of “pathomics” has gained significant interest. Pathomics encompasses a broad range of data extracted from digital pathology image analysis. This approach quantifies features to describe various tissue sample phenotypes, and the resulting data is employed for diagnostic purposes or to predict survival outcomes [14, 15, 16]. In this context, we propose that pathomics analysis of digital H&E‐stained images could be instrumental in ALN metastasis (ALNM) prediction in BC patients.

Our study aims to develop a nomogram model that integrates both clinicopathological variables and whole slide images (WSIs) to predict ALNM status. This model has the potential to guide healthcare professionals in tailoring treatment strategies, ultimately enhancing the effectiveness of personalized therapeutic approaches.

## 2 Materials and Methods

### 2.1 Participants

**FIGURE 1:**
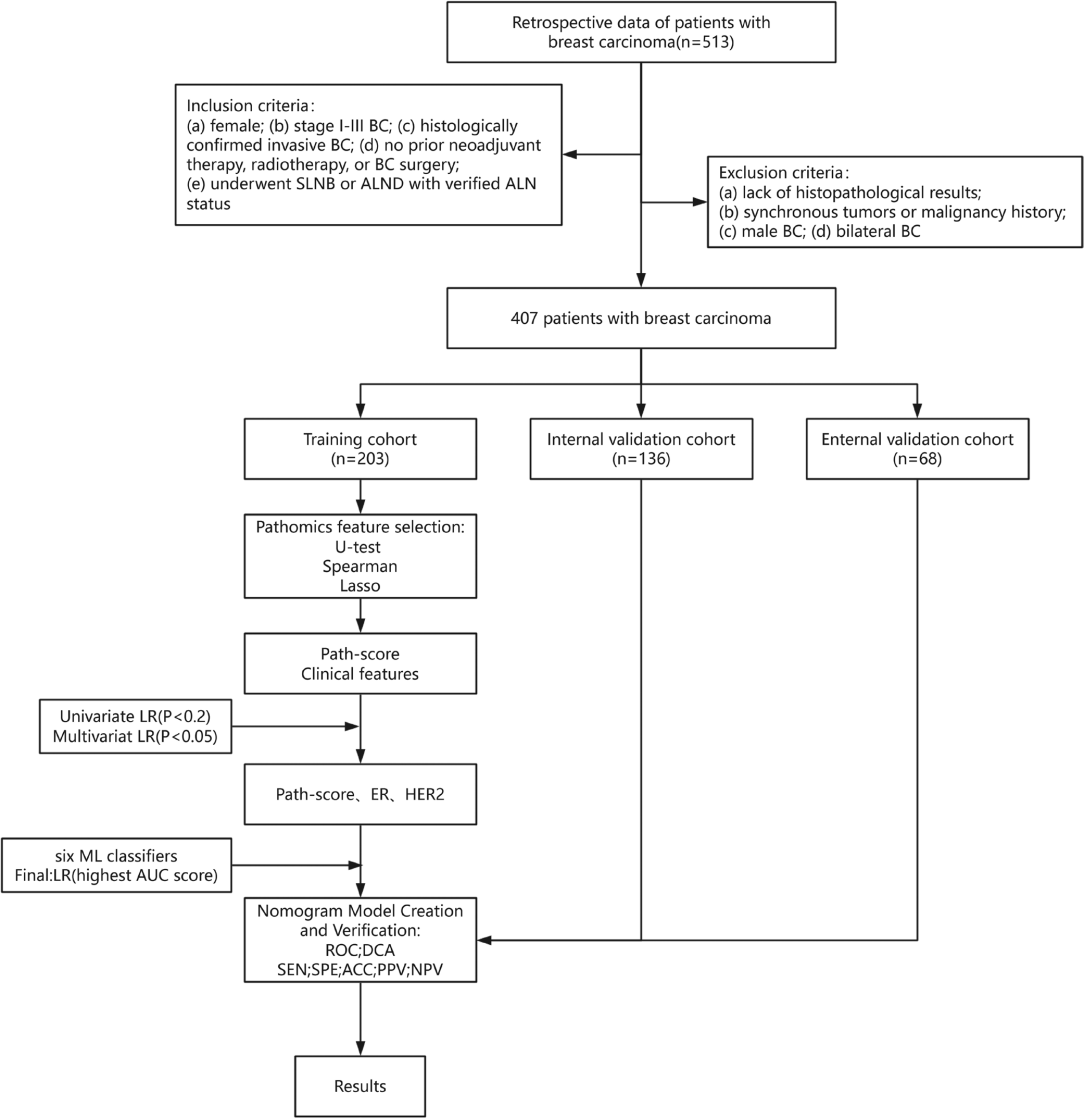
Flowchart of the overall construction process of the machine learning models predicting ALNM status. ALNM, axillary lymph node metastasis; IVC, internal validation cohort; EVC, external validation cohort; TC, training cohort.

This retrospective study was conducted on 407 patients diagnosed with primary BC at two medical institutions: the First Affiliated Hospital of Chongqing Medical University and Chongqing University Cancer Hospital. The patients included in this study were hospitalized between January 2019 and December 2023. The patients met the following inclusion criteria: (a) female gender; (b) Stages I–III BC; (c) histologically confirmed invasive BC; (d) no prior neoadjuvant therapy, radiotherapy, or breast surgery; and (e) SLNB or ALND with confirmed ALN status. The exclusion criteria were: (a) lack of histopathological results; (b) synchronous tumors or malignancy history; (c) male BC; and (d) bilateral BC. ALN‐positive status was defined as the presence of tumor cells in at least one ALN, which could include macrometastases, micrometastases, or isolated tumor cells (ITCs) [17]. The initial cohort was split into a training cohort (TC; *n* = 203) and an internal validation cohort (IVC; *n* = 136) at a 6:4 ratio. An additional external validation cohort (EVC; *n* = 68) was recruited from patients with BC at the First Affiliated Hospital of Chongqing Medical University. Ki67 expression was categorized into two groups: low (< 14%) and high (≥ 14%) [18, 19, 20]. A detailed flowchart illustrating the study methodology is presented in Figure 1.

### 2.2 Sample Preparation and Region of Interest (ROI) Selection

Biopsy samples from BC patients were obtained by pathologists through coarse needle aspiration. The biopsy specimens were initially immersed in 10% formalin for 4 h and then embedded in paraffin wax for immunohistochemical analysis. The tissues were subsequently sectioned at 4‐μm intervals and stained with H&E for pathological examination. An expert pathologist with 7 years of diagnostic experience used a digital scanner to digitize the H&E‐stained slides, creating WSIs for analysis.

For each case, two pathologists independently identified seven non‐overlapping representative tiles with the highest concentration of tumor cells, each with a resolution of 1000 × 1000 pixels. Areas containing tissue folds were excluded. Afterward, we saved the chosen tiles as .tif files. In cases of disagreement, a third pathologist was consulted to reach a consensus.

### 2.3 Pathomics Feature Extraction

CellProfiler (v4.2.6), a free image analysis software from the Broad Institute, was used to extract quantitative pathomics features from pathological images [21]. To achieve this, the “Unmix Colors” module separated the H&E‐stained images into individual grayscale images for H&E. In addition, the “ColorToGray” module was used to create a grayscale version of the original H&E image. This process resulted in the extraction of 1121 pathomics features. The features were then summarized by calculating the mean value across all seven tiles within each WSI.

### 2.4 Feature Selection and Signature Construction

Although pathomics features offer valuable insights into tumor characteristics from a microenvironmental perspective, the high dimensionality of this data poses challenges for accurately predicting ALNM. To overcome this hurdle, we employed a feature selection process, aiming at identifying the most relevant features within the TC. After normalizing the features, a *U*‐test was conducted on each feature to preliminarily filter out redundant ones, with a *p* value threshold of 0.05 set for statistical significance. Additionally, a correlation analysis was conducted to account for potential inter‐feature dependencies. Our study excluded any two features with a correlation coefficient > 0.9 to avoid redundancy.

Subsequently, the least absolute shrinkage and selection operator (LASSO) algorithm was employed to choose features, with the Lambda value determined through 10‐fold cross‐validation to determine the best features. Finally, we calculated a pathomics score (Path‐score) for each lesion via LASSO regression. This score combined the chosen features weighted by their nonzero coefficients.

### 2.5 ML Classifier Construction

For feature selection, we included features from both clinical characteristics and the Path‐score that demonstrated a *p* < 0.2 in the ULR analysis while utilizing a stricter threshold of *p* < 0.05 in the MLR analysis to determine statistical significance. These selected features were then employed to build a prediction model in the TC. Six ML classifiers were utilized: decision tree (DT), random forest (RF), K‐nearest neighbor (KNN), light gradient boosting machine (LGBM), logistic regression (LR), and XGBoost (XGB). Eventually, we selected the classifier with the highest AUC score in the IVC as the final model.

### 2.6 The Nomogram Model Creation and Verification

After identifying the statistically significant features (Path‐score and clinical characteristics) that differentiate between ALN‐positive and ALN‐negative BC cases in the TC, we utilized logistic regression analysis to build prediction models. The models' performance was assessed in the IVC and EVC employing metrics including receiver operating characteristic (ROC) curve, accuracy (ACC), positive predictive value (PPV), negative predictive value (NPV), sensitivity (SEN), and specificity (SPE). Calibration curves were plotted to assess model reliability, and decision curve analysis (DCA) was employed to identify models that provide the greatest clinical benefit for patients. Finally, a pathomics nomogram model incorporating these significant clinical variables alongside the Path‐score was developed for individualized prediction of ALNM.

### 2.7 Statistical Analysis

Statistical analysis was conducted using R Studio (v4.1.3) and Jupyter Notebook (v6.4.11). Categorical variables were compared using either the chi‐square test or Fisher's exact test, depending on the sample size. The analysis of continuous variables was performed employing either the independent *t*‐test or the Mann–Whitney *U*‐test according to data normality. All tests were two‐sided, with *p* < 0.05 deeming a significant level.

## 3 Results

### 3.1 Clinical and Pathological Characteristics

Based on the predefined inclusion and exclusion criteria, 339 bc patients from Chongqing University Cancer Hospital were randomly assigned to the TC (*n* = 203) and the validation cohort (*n* = 136) in a 6:4 ratio. An additional EVC of 68 patients was recruited from the First Affiliated Hospital of Chongqing Medical University (Table 1).

**TABLE 1:** Baseline clinicopathological characteristics in the training cohort (TC) and validation cohorts.

Characteristics	Overall (*n* = 339)	TC (*n* = 203)	Internal validation cohort (*n* = 136)	*p*	External validation cohort (*n* = 68)	*p*
Age (years)	53.00 [48.00–59.00]	53.00 [48.00–5 8.00]	53.00 [48.00–59.00]	0.904	52.85 (11.66)	0.633
BMI	24.60 [22.70–26.80]	24.40 [22.75–26.60]	25.10 [22.70–27.30]	0.252	24.27 [22.30–25.83]	0.234
Menopause				0.972		0.401
No	123 (36.28%)	73 (35.96%)	50 (36.76%)		29 (42.65%)	
Yes	216 (63.72%)	130 (64.04%)	86 (63.24%)		39 (57.35%)	
Tumor family history				0.232		1.000
No	291 (85.84%)	170 (83.74%)	121 (88.97%)		57 (83.82%)	
Yes	48 (14.16%)	33 (16.26%)	15 (11.03%)		11 (16.18%)	
ER				0.804		0.127
Negative	91 (26.84%)	53 (26.11%)	38 (27.94%)		25 (36.76%)	
Positive	248 (73.16%)	150 (73.89%)	98 (72.06%)		43 (63.24%)	
PR				0.840		0.751
Negative	161 (47.49%)	95 (46.80%)	66 (48.53%)		34 (50.00%)	
Positive	178 (52.51%)	108 (53.20%)	70 (51.47%)		34 (50.00%)	
HER2				0.799		0.133
Negative	253 (74.63%)	150 (73.89%)	103 (75.74%)		57 (83.82%)	
Positive	86 (25.37%)	53 (26.11%)	33 (24.26%)		11 (16.18%)	
Ki67 expression (%)				0.390		1.000
< 14	54 (15.93%)	29 (14.29%)	25 (18.38%)		10 (14.71%)	
≥ 14	285 (84.07%)	174 (85.71%)	111 (81.62%)		58 (85.29%)	
Tumor size				0.820		0.079
T1	64 (18.88%)	36 (17.73%)	28 (20.59%)		14 (20.59%)	
T2	229 (67.55%)	137 (67.49%)	92 (67.65%)		41 (60.29%)	
T3	18 (5.31%)	12 (5.91%)	6 (4.41%)		10 (14.71%)	
T4	28 (8.26%)	18 (8.87%)	10 (7.35%)		3 (4.41%)	
Molecular subtypes				0.851		0.098
Luminal	195 (57.52%)	115 (56.65%)	80 (58.82%)		37 (54.41%)	
Luminal/HER2	54 (15.93%)	35 (17.24%)	19 (13.97%)		6 (8.82%)	
HER2	32 (9.44%)	18 (8.87%)	14 (10.29%)		5 (7.35%)	
TNBC	58 (17.11%)	35 (17.24%)	23 (16.91%)		20 (29.41%)	
Path_score	−0.37 [−0.59 to −0.10]	−0.34 [−0.57 to −0.07]	−0.41 [−0.65 to −0.16]	0.092	−0.46 [−0.62 to −0.08]	0.221

Abbreviations: BMI, body mass index; ER, estrogen receptor; HER2, human epidermal growth factor receptor 2; Ki67, cell proliferation marker; PR, progesterone receptor.

Among the participants, 123 (36.28%) were premenopausal, while the remaining 216 (63.72%) were postmenopausal. The majority (85.84%, *n* = 291) reported no family history of cancer. ER positivity was observed in 73.16% of patients, and 52.51% (*n* = 178) were PR positive. HER2 negativity was found in 253 (74.63%) patients, and a significant portion (84.07%, *n* = 285) exhibited Ki67 expression ≥ 14%. Regarding tumor size distribution, 67.55% (*n* = 229) were classified as T2, 18.88% (*n* = 64) as T1, 8.26% (*n* = 28) as T4, and 5.31% (*n* = 18) as T3. Notably, there were no significant differences in clinicopathological characteristics between the training and validation cohorts.

### 3.2 Pathomics and Clinical Feature Selection

**FIGURE 2:**
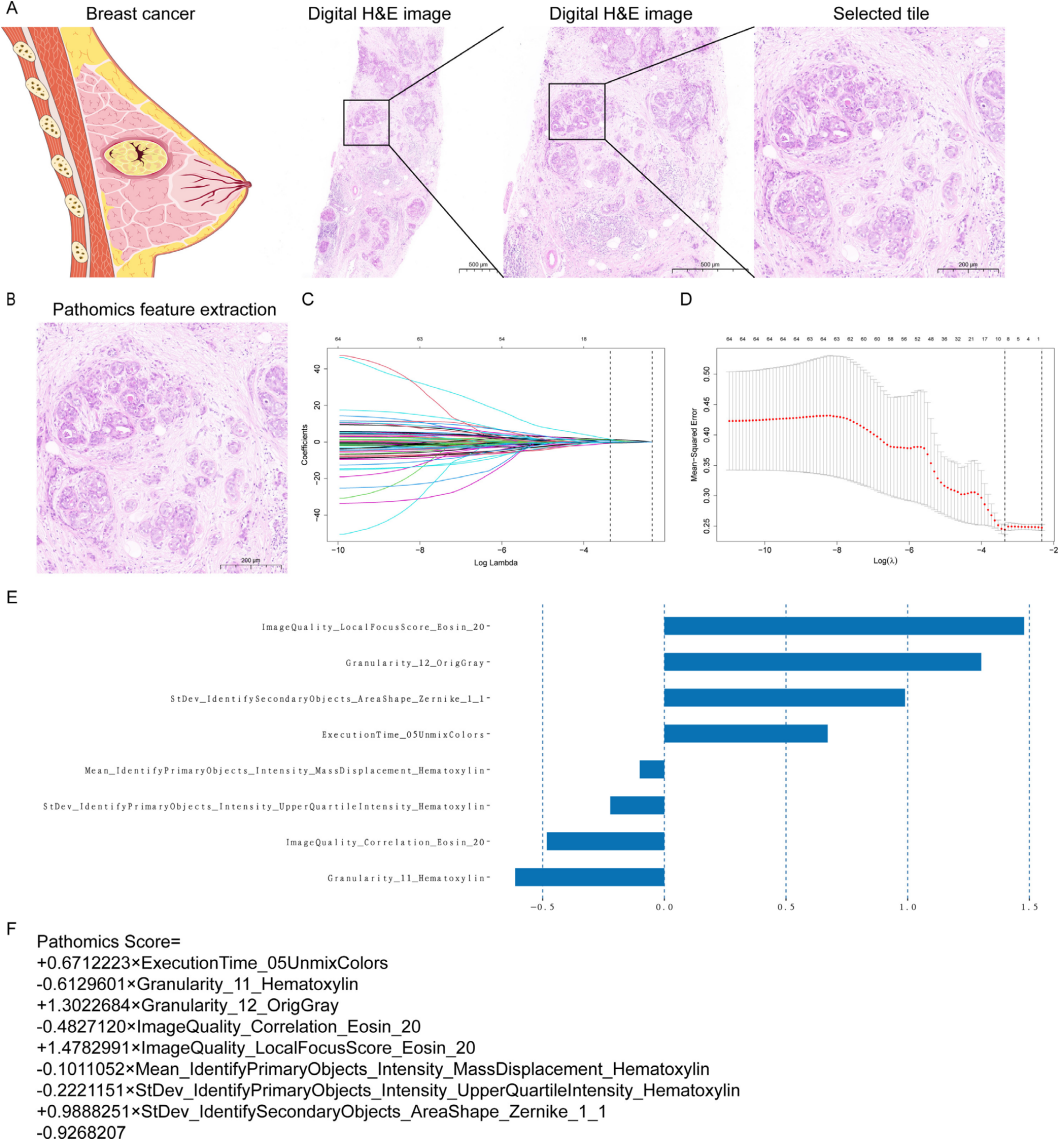
Schematic illustration of the constructed pathomics signature. (A) Representative H&E tile selection. Scale bar: 500, 500, and 200 μm, respectively. (B) The usage of the chosen tile to extract the pathomics feature. Selection of the pathomics feature through (C) LASSO binary regression with (D) 10‐fold cross‐validation. (E) The pathomics feature coefficients are used to construct the Path‐score. (F) Calculation of the pathomics signature relying upon the chosen features. H&E, hematoxylin and eosin; LASSO, least absolute shrinkage and selection operator.

CellProfiler was used to extract 1121 pathomics features from WSIs. Redundant features were removed through a two‐step process: first, a *U*‐test was conducted to identify features without significant differences between groups, followed by a Spearman correlation analysis to eliminate highly interdependent features. This resulted in a set of 64 unique pathomics features per patient (Figure 2A,B). The LASSO technique was then employed for further feature selection. This method identified a subset of the most informative features, leading to the creation of an eight‐feature pathomics signature. The signature was derived through LASSO logistic regression with 10‐fold cross‐validation to optimize model performance (Figure 2C–E).

**FIGURE 3:**
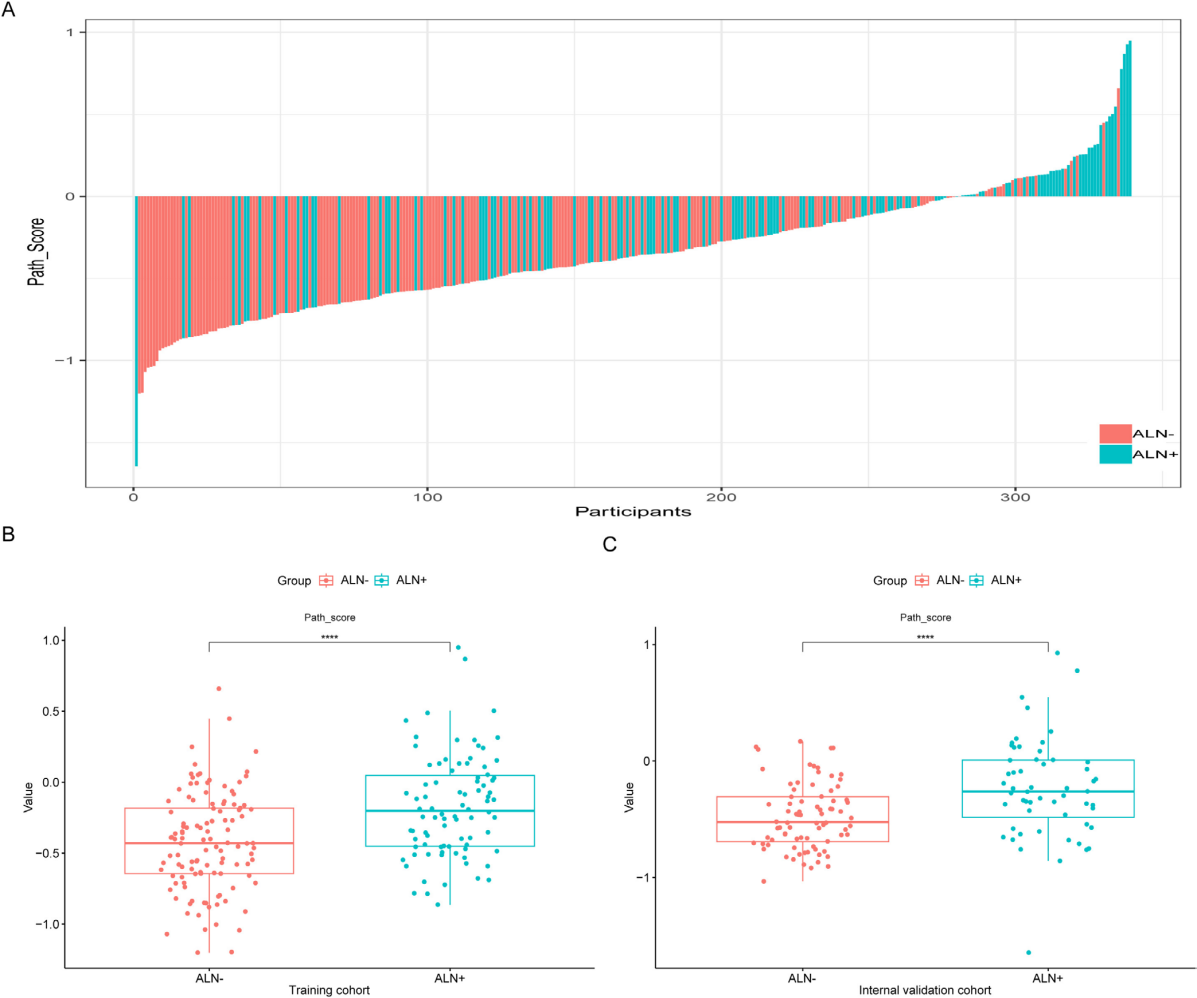
The distribution of pathological scores among all patients and across cohorts. (A) Pathomics score for each patient suffering from BC in the total cohort; distribution of pathomics score values of the ALN+/ALN− groups in the TC (B) and IVC (C). ALN, axillary lymph node; BC, breast cancer; IVC, internal validation cohort; TC, training cohort.

Our study calculated the Path‐score as a linear combination of the nonzero coefficient features identified through the LASSO model (Figure 2F). Notably, the Path‐score distribution (median [interquartile range]) displayed good comparability between the TC (−0.34 [−0.57 to −0.07]), IVC (−0.41 [−0.65 to −0.16]), and EVC (−0.46 [−0.62 to −0.08]) (Table 1). Figure 3A illustrates the Path‐score distribution for individual patients. Within the TC, the Path‐score medians showcased a statistically significant difference between patients having positive (ALN+) and negative (ALN−) ALN status (*p* < 0.001) (Figure 3B). This finding was replicated in the IVC (*p* < 0.001) (Figure 3C).

ULR analysis was conducted to evaluate each variable's influence on the ALNM risk in BC. Variables having *p* < 0.2 were considered for further analysis. As Table 2 summarizes, ER status (*p* = 0.003), PR status (*p* = 0.002), HER2 status (*p* = 0.017), Ki67 expression (*p* = 0.023), and tumor size (*p* = 0.102) all exhibited significant correlations with ALNM. The Path‐score demonstrated the strongest association (*p* < 0.001). Conversely, other variables were not significantly correlated with ALNM. Following the ULR and MLR analyses, it was revealed that ER status (OR = 2.692, 95% CI = [1.277–5.985], *p* = 0.011), HER2 status (OR = 0.383, 95% CI = [0.176–0.796], *p* = 0.012), tumor size (OR = 1.538, 95% CI = [1.023–2.373], *p* = 0.043), and Path‐score (OR = 7.374, 95% CI = [3.014–19.68], *p* < 0.001) remained significantly related to ALNM.

**TABLE 2:** Univariate (ULR) and multivariate (MLR) logistic regression analyses of axillary lymph node metastasis predictors in the training cohort.

Characteristics	ULR analysis			MLR analysis		
	OR	95% CI	*p*	OR	95% CI	*p*
Age	1.011	1.011 (0.983–1.04)	0.442			
Body mass index	1.049	1.049 (0.958–1.15)	0.305			
Menopause	0.994	0.994 (0.557–1.781)	0.983			
Tumor family history	1.162	1.162 (0.543–2.459)	0.695			
Estrogen receptor	2.917	2.917 (1.477–6.078)	0.003	2.692	2.692 (1.277–5.985)	0.011
Progesterone receptor	2.544	2.544 (1.437–4.572)	0.002			
Human epidermal growth factor 2	0.439	0.439 (0.218–0.851)	0.017	0.383	0.383 (0.176–0.796)	0.012
Ki67 expression	0.392	0.392 (0.17–0.87)	0.023			
Tumor size	1.358	1.358 (0.944–1.978)	0.102	1.539	1.538 (1.023–2.373)	0.043
Pathomics score	7.743	7.743 (3.272–19.82)	< 0.001	7.374	7.374 (3.014–19.68)	< 0.001

Abbreviations: 95% CI, 95% confidence interval; OR, odds ratio.

### 3.3 Construction and Evaluation of ML Classifier

**FIGURE 4:**
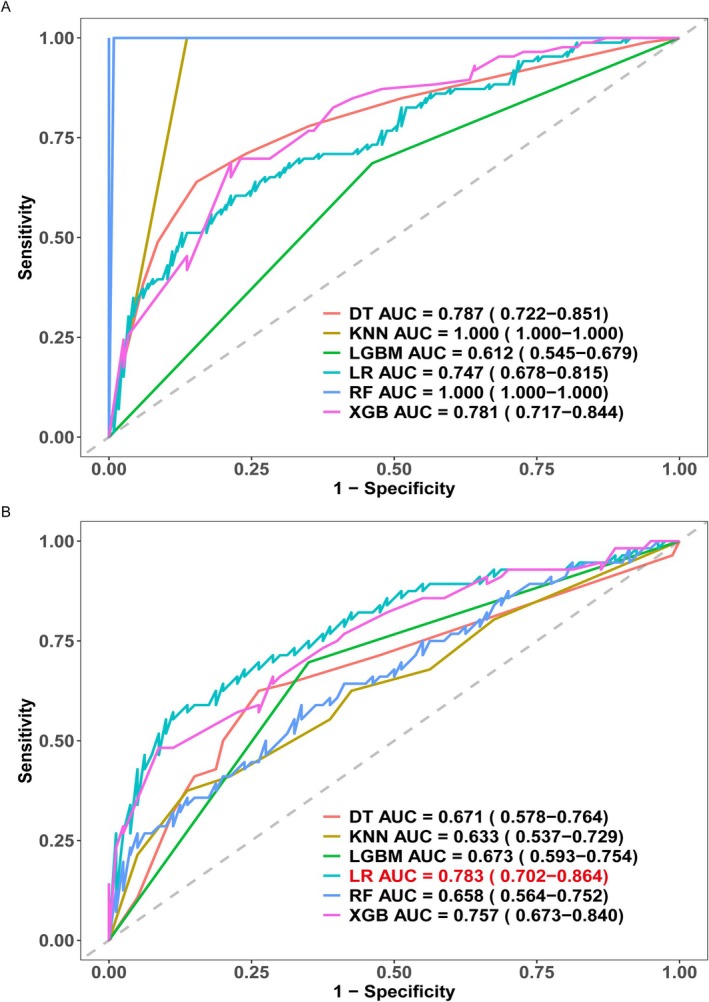
ROC curves of the six machine learning classifiers that predict the ALNM in the TC (A) and IVC (B). ALNM, axillary lymph node metastasis; IVC, internal validation cohort; ROC, receiver operating characteristic; TC, training cohort.

Six ML classifiers (DT, LR, RF, XGBoost, KNN, and LGBM) were employed to build prediction models. The Path‐score and clinical features served as input variables for these models. Figure 4 compares the ROC curves for all six classifiers in both the TC and IVC. The results suggested potential overfitting in the RF and KNN classifiers. Although these models achieved perfect AUC scores of 1.000 in the TC, their performance significantly decreased in the IVC (AUC = 0.658 and 0.633, respectively). In the IVC, the AUC values for the six models were 0.633–0.783, with the LR model demonstrating the strongest performance and the KNN model performing the weakest. Using the DeLong test, the statistical significance of the AUC differences was assessed between each pair of classifiers within the IVC (Table 3). The final nomogram model was selected based on the LR classifier, which achieved the highest AUC value.

**TABLE 3:** *p* values for area under the curve (AUC) comparison between any pair of models examined by the DeLong test in the internal validation cohort.

Model (AUC value)	Decision tree (DT) (**0.671**)	Logistic regression (LR) (**0.783**)	Random forest (RF) (**0.658**)	Extreme gradient boosting (XGBoost) (**0.757**)	K nearest neighbor (KNN) (**0.633**)	Light gradient boosting machine (LGBM) (**0.673**)
DT (**0.671**)	1	0.405549269	9.56434E−11	0.892846002	9.56434E−11	0.000270527
LR (**0.783**)	0.405549269	1	8.24604E−12	0.477815301	8.24604E−12	0.006157342
RF (**0.658**)	9.56434E−11	8.24604E−12	1	1.08228E−10	1	2.19396E−23
XGBoost (**0.757**)	0.892846002	0.477815301	1.08228E−10	1	1.08228E−10	1.16585E−06
KNN (**0.633**)	9.56434E−11	8.24604E−12	1	1.08228E−10	1	2.19396E−23
LGBM (**0.673**)	0.000270527	0.006157342	2.19396E−23	1.16585E−06	2.19396E−23	1

*Note:* The bold numbers (< 0.05) mean statistical difference.

### 3.4 The Nomogram Model Development and Validation

**FIGURE 5:**
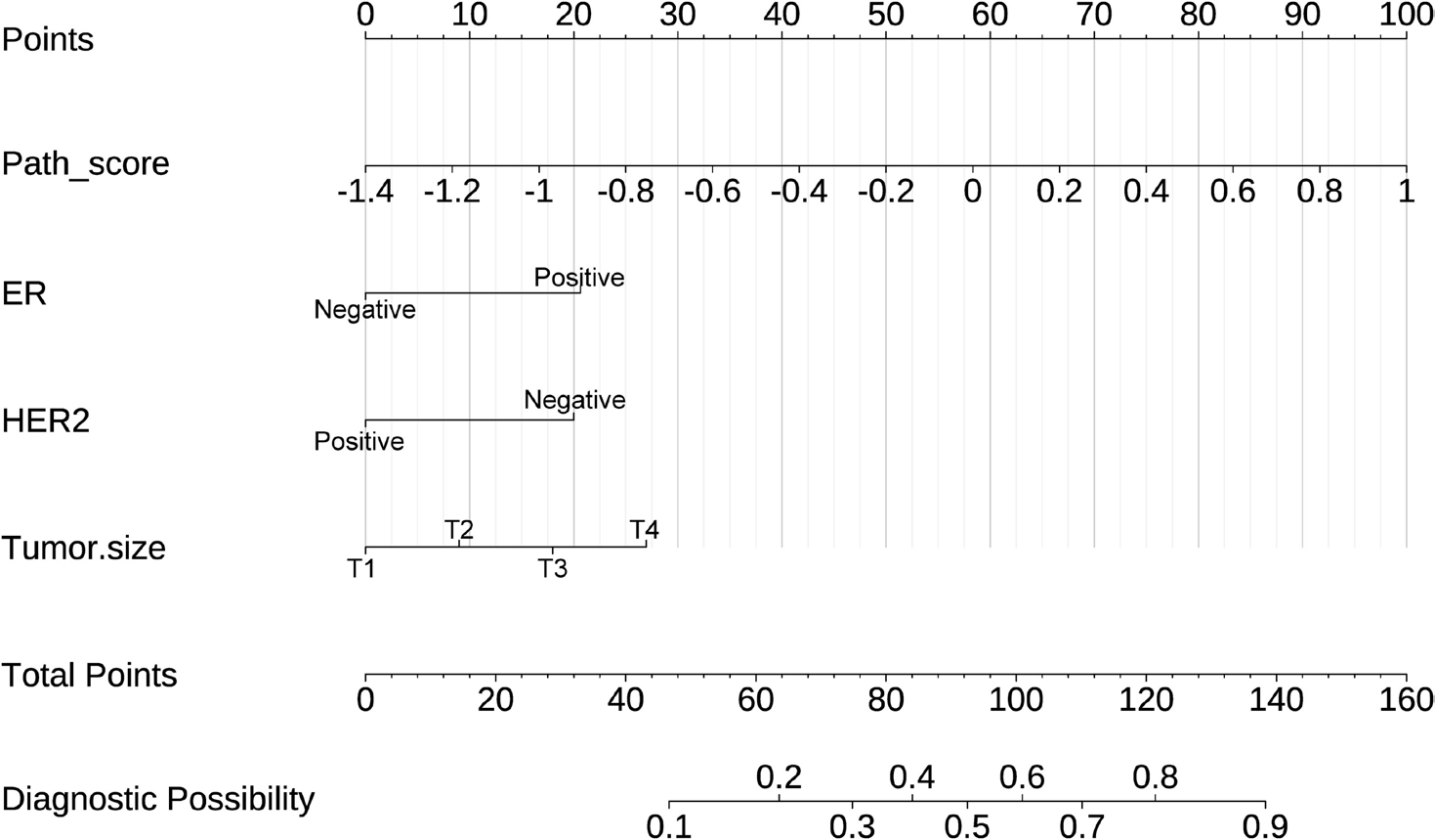
The developed nomogram based on the combined ER, HER2, tumor size, and Path‐score through logistic regression analysis. ER, estrogen receptor; HER2, human epidermal growth factor receptor 2.

**FIGURE 6:**
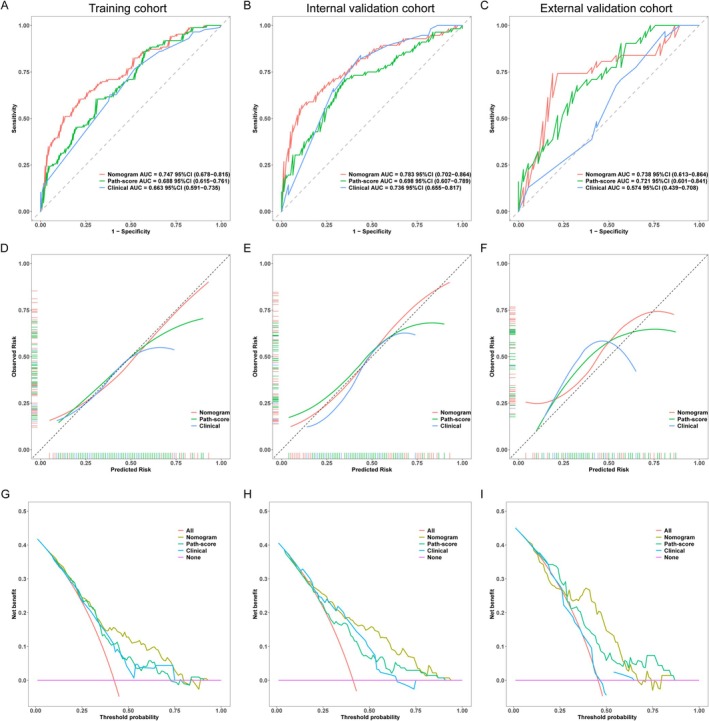
Validation of the Path‐score‐based nomogram predictive value. The nomogram model's ROC curves in the (A) TC, (B) IVC, and (C) EVC. The nomogram model calibration plots in the (D) TC, (E) IVC, and (F) EVC. The net benefit of nomogram usage is shown by the decision curves in the (G) TC, (H) IVC, and (I) EVC. EVC, external validation cohort; IVC, internal validation cohort; ROC, receiver operating characteristic; TC, training cohort.

To predict ALNM, a combined nomogram incorporating four independent predictors was developed: ER status, HER2 status, tumor size, and Path‐score. The nomogram utilizes the regression coefficients of these variables to calculate a total score, with individual points assigned to each variable on the basis of its contribution. The nomogram assigns points to each factor using a point scale (Figure 5). These points are then combined to predict ALNM probability. The nomogram model performance was compared to the path‐score model and a model based solely on clinical features (Table 4). Within the IVC, the nomogram achieved the highest AUC of 0.783, outperforming the path‐score model (AUC = 0.698) and significantly surpassing the clinical model (AUC = 0.736). This superior performance was replicated in the EVC, where the nomogram elucidated an AUC of 0.738 in comparison to 0.721 for the path‐score model and 0.574 for the clinical model. Pairwise DeLong tests (Table 5) confirmed the statistical significance of these differences. Notably, the nomogram's AUC significantly differed from the path‐score model in the IVC (AUC, 0.783 vs. 0.698; DeLong test, *p* = 0.008558) and from the clinical model in the EVC (AUC, 0.738 vs. 0.574; DeLong test, *p* = 0.00494). Figure 6A–C illustrates the three models' ROC curves in ALNM prediction.

**TABLE 4:** Predictive performances of the models predicting the axillary lymph node metastasis status in patients having breast cancer.

Model	Cohorts	Area under the curve (95% CI)	Sensitivity (%)	Specificity (%)	Accuracy (%)	Positive predictive value (%)	Negative predictive value (%)
Nomogram	Training cohort (TC)	0.747 (0.678–0.815)	0.605	0.786	0.709	0.675	0.73
	Internal validation cohort (IVC)	0.783 (0.702–0.864)	0.571	0.887	0.757	0.78	0.747
	External validation cohort (EVC)	0.738 (0.613–0.864)	0.742	0.811	0.779	0.767	0.789
Pathomics score	TC	0.688 (0.615–0.761)	0.605	0.692	0.655	0.591	0.704
	IVC	0.698 (0.607–0.789)	0.714	0.65	0.676	0.588	0.765
	EVC	0.721 (0.601–0.841)	0.677	0.676	0.676	0.636	0.714
Clinical	TC	0.663 (0.591–0.735)	0.767	0.479	0.601	0.52	0.737
	IVC	0.736 (0.655–0.817)	0.839	0.562	0.676	0.573	0.833
	EVC	0.574 (0.439–0.708)	0.968	0.189	0.544	0.5	0.875

**TABLE 5:** DeLong test results of the model comparison in the prediction of the axillary lymph node metastasis status in patients having breast cancer.

Model comparison	Training cohort (*p*)	Internal validation cohort (*p*)	External validation cohort (*p*)
Nomogram vs. Pathomics score	0.03869	0.008558	0.6992
Nomogram vs. Clinical	0.004817	0.1802	0.00494

*Note:* DeLong test, statistical test for comparing AUC values.

**FIGURE 7:**
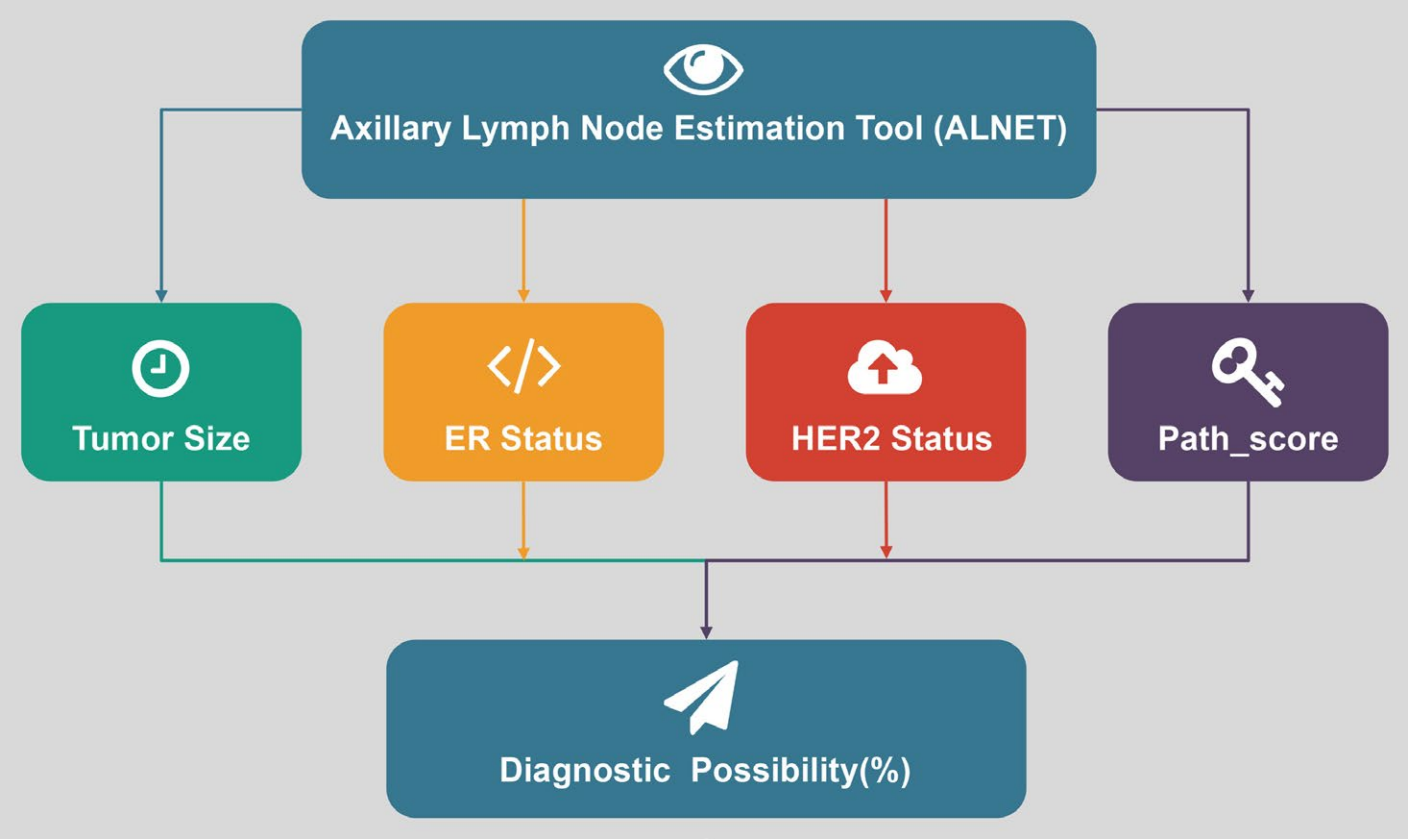
The ALNET: A simple program interface that predicts ALNM based on tumor size, ER status, HER2 status, and Path‐score. ALNET, Axillary Lymph Node Estimation Tool; ALNM, axillary lymph node metastasis; ER, estrogen receptor; HER2, human epidermal growth factor receptor 2.

Calibration, which measures how well a model's predicted probabilities align with actual outcomes, was assessed using the Hosmer‐Lemeshow test (Table 6). Comparable results (*p* > 0.05) were obtained in all cohorts (TC: *p* = 0.6613, IVC: *p* = 0.4464, EVC: *p* = 0.1314), indicating good model calibration (Figure 6D–F). Herein, we deployed DCA [22] to compare the nomogram clinical benefit, Path‐score, and clinical models. As shown in Figure 6G–I, across a range of predicted probability thresholds (20%–80%), decisions based on the nomogram model were more beneficial for patients compared to treating all or none of them. To enhance the clinical application of the nomogram, we developed a mini‐program that allows real‐time input of key predictors—ER status, HER2 status, tumor size, and Path‐score—to estimate ALNM probability. The interface simplifies the use of the nomogram by offering instant predictions without complex calculations (Figure 7). This digital tool improves accessibility and practicality, making the model more user‐friendly for clinical decision‐making.

**TABLE 6:** Hosmer–Lemeshow test results of the nomogram model's calibration in the three cohorts of breast cancer patients.

Model comparison	Training cohort (*p*)	Internal validation cohort (*p*)	External validation cohort (*p*)
Nomogram	0.6613	0.4464	0.1314

*Note:* Hosmer–Lemeshow test, statistical test for model calibration.

## 4 Discussion

Accurate prediction of ALNM is essential for guiding surgical decisions in BC patients. Our study constructed and verified a pathomics‐based nomogram for ALNM prediction. Six ML models were evaluated using the Path‐score and clinical features in the training and IVC. The LR model showed the highest AUC of 0.783 in the IVC and was selected for the final nomogram. Our retrospective analysis revealed a significant association between the pathomics signature and ALNM. Furthermore, the developed and validated nomogram demonstrated reliable discrimination and calibration for ALNM prediction in BC patients.

ALN status constitutes a critical factor in BC diagnosis, guiding treatment decisions. Patients who have an ALN+ status often have worse outcomes in comparison to those who have an ALN− status [23, 24, 25]. Therefore, accurate assessment of both the primary tumor and ALN involvement is essential, both before and after NAC [26]. However, it is important to note that while our nomogram can assist in preoperative assessments, its application in NAC‐treated patients should be approached with caution [17]. NAC can induce a complete pathological response (pCR), but pathological evaluation of surgically removed lymph nodes remains necessary to detect micrometastases or ITCs, which have significant prognostic value [27]. Thus, our nomogram is more suitable for patients who are candidates for upfront surgery rather than those undergoing NAC.

Preoperative assessment of ALNM primarily relies on noninvasive imaging techniques like mammography, ultrasound, MRI, and PET/CT [28, 29]. However, these methods have limitations in diagnostic accuracy. Increasing interest has emerged in artificial intelligence (AI)‐based solutions for analyzing tumor heterogeneity. ML approaches using radiomics features have dominated ALNM prediction research. Yet, their accuracy is hindered by variability in specificity and operator dependency, particularly for detecting subtle ALN abnormalities.

The integration of digital pathology with AI has significantly advanced tumor research, offering new opportunities to improve cancer diagnosis, prognosis, and treatment. Advancements in slide scanning technology and affordable digital storage have enabled the conversion of stained tissue sections into high‐resolution digital formats. Coupled with AI technologies like deep learning, this transformation has enhanced the visualization and analysis of pathological images [30]. Convolutional neural networks (CNNs) automate tumor cell recognition and classification, reducing errors in manual diagnoses [31]. AI has also proven valuable in predicting patient outcomes and identifying biomarkers, thereby advancing personalized treatment strategies [32]. Additionally, pathomics allows for a deeper exploration of the TME, a critical factor in cancer progression and metastasis [33]. AI‐driven models can detect subtle changes in TME that are often missed through manual analysis, aiding in treatment response predictions. In our study, the pathomics‐based nomogram demonstrated high predictive accuracy for ALNM. However, it should be viewed as a clinical decision support tool rather than a standalone predictor. Clinicians should use the nomogram alongside other diagnostic tools, particularly when pathological confirmation is necessary, to avoid overextending its clinical application.

Our study identified ER status, HER2 status, tumor size, and Path‐score as independent predictors of ALNM using both ULR and MLR analyses. Previous research has established the critical roles of ER, HER2, and tumor size in BC ALNM [34, 35]. ER, HER2, and tumor size are well‐established markers in BC management, with critical roles in predicting lymph node involvement and guiding therapeutic decisions [36]. In the early stages of the study, we considered a broader set of clinical variables such as age, BMI, Ki67, menopausal status, tumor family history, and other clinical features. These variables were systematically narrowed through ULR and MLR, ensuring that only the most relevant predictors were included in the final model. This approach ensured that the model remained practical without overcomplicating the prediction process. However, the value of pathomics scores for predicting ALNM has been less explored. This study's innovation lies in the development of a pathomics nomogram that integrates both macroscopic clinical features and microscopic pathomics information. This comprehensive approach offers the potential for ALNM prediction in BC patients, potentially reducing unneeded treatments. Nonetheless, as our results suggest, caution should be taken in interpreting the nomogram's predictions, especially in the context of highly variable tumor biology and intratumoral heterogeneity, which can lead to different biomarker profiles between primary tumors and metastatic sites.

Our pathomics‐based nomogram offers several clinical advantages, including simplicity, stability, reproducibility, and cost‐effectiveness. The model begins by applying LASSO regression to generate the Path‐score from key pathomic features, followed by univariate and multivariate logistic regression to refine clinical variables such as ER status, HER2 status, and tumor size for inclusion in the final model. This ensures that the model is robust and easy to implement in clinical practice without the need for expensive or complex testing. LASSO regression improves the model's generalizability by minimizing overfitting, while its reliance on routine clinical data and digital pathology images enhances its cost‐effectiveness by reducing the need for costly diagnostics [37]. ROC curve analysis was utilized to determine the cutoff values, balancing sensitivity and specificity [38]. Although internal validation revealed lower sensitivity, similar limitations have been observed in other predictive models that rely on multiple indicators [39]. Therefore, our nomogram should be seen as a clinical decision support tool rather than a standalone diagnostic. To further enhance accessibility, we developed a mini‐program (Figure 7) that allows clinicians to input these key variables—ER status, HER2 status, tumor size, and Path‐score—and instantly calculate ALNM probability, simplifying its application in clinical settings. By combining the model with other diagnostic methods, such as imaging and histopathological evaluation, the risk of missed diagnoses can be mitigated. Further prospective validation in larger and more diverse populations is essential to refine the model's sensitivity and broaden its clinical applicability, as recent studies have underscored the importance of validating AI‐driven models across various patient groups [40].

While our study is pioneering, it is important to acknowledge its limitations. First, it is a two‐center retrospective investigation with a limited sample size. This necessitates further research with a larger and more varied group of patients from several institutions. Prospective studies should verify the generalizability and accuracy of the pathomics nomogram. Secondly, the current manual segmentation of the ROI in tumors is time‐consuming. Future advancements in AI‐driven automated segmentation hold promise in addressing this challenge. Additionally, further investigation into the prognostic relevance of micrometastases and ITCs, which our nomogram does not directly address, could refine its predictive power and clinical utility.

## 5 Conclusion

In conclusion, this study identified ER status, HER2 status, tumor size, and Path‐score as independent predictors of ALNM in BC patients using both univariate and multivariate logistic regression analyses. The pathomics‐based nomogram demonstrated high accuracy in the prediction of ALNM probability, thereby aiding in the development of personalized treatment plans. These findings underscore the potential of AI to advance precision medicine approaches in BC management.

## Author Contributions

**Long Wang:** data curation (equal), formal analysis (equal), writing – original draft (lead). **Fanli Qu:** formal analysis (equal). **Ping Wen:** data curation (equal). **Yu Luo:** data curation (equal). **Huan Zhang:** data curation (equal). **Shanqi Li:** project administration (equal). **Xuedong Yin:** project administration (equal). **Yulan Zhao:** investigation (equal), writing – review and editing (equal). **Xiaohua Zeng:** investigation (equal), writing – review and editing (equal).

## Ethics Statement

The Institutional Ethics Committees of the First Affiliated Hospital of Chongqing Medical University (K2023‐571) and Chongqing University Cancer Hospital (CZLS2024092‐A) approved this investigation, which adhered to the Helsinki Declaration.

## Conflicts of Interest

The authors declare no conflicts of interest.

## Data Availability

The used data sets can be accessed upon reasonable request from the corresponding author.
